# Laparoscopic lavage versus resection in perforated diverticulitis with purulent peritonitis: a meta-analysis of randomized controlled trials

**DOI:** 10.1186/s13017-016-0103-4

**Published:** 2016-08-30

**Authors:** Marco Ceresoli, Federico Coccolini, Giulia Montori, Fausto Catena, Massimo Sartelli, Luca Ansaloni

**Affiliations:** 1 Unit of General and Emergency Surgery, Papa Giovanni XXIII Hospital, Piazza OMS 1, 24127 Bergamo, Italy; 2 Unit of General and Emergency Surgery, Parma University Hospital, Parma, Italy; 3 Unit of General and Emergency Surgery, Macerata Hospital, Macerata, Italy

**Keywords:** Acute diverticulitis, Purulent peritonitis, Acute perforated diverticulitis, Laparoscopic lavage, Meta-analysis

## Abstract

**Objective:**

Purulent peritonitis from acute left colon diverticulitis is a relatively common presentation of diverticular disease; historically the treatment was the Hartmann procedure. Laparoscopic peritoneal lavage has been proposed as a lesser invasive treatment option with great interest and debate among surgeons and with contrasting results. The aim of this meta-analysis was to compare the results of sigmoid resection with laparoscopic lavage.

**Methods:**

A systematic review was performed to select randomized controlled trials comparing laparoscopic lavage versus resection in Hinchey III diverticulitis. Studies’ selection, data extraction and risk of bias assessment were done by two independent authors; results were shown as OR with 95 % C.I.

**Results:**

Three RCT were selected for the meta-analysis including 315 patents. Laparoscopic lavage was associated with significantly more reoperations (OR 3.75, *p* = 0.006) and more intra-abdominal abscesses (OR 3.50, *p* = 0.0003) with no differences in mortality (OR 0.93, *p* = 0.92). At 12 months follow up laparoscopic lavage was associated with lesser reoperations (OR 0.32, *p* = 0.0004); there were no differences in term of stoma presence (OR 0.44 *p* = 0.27) and mortality (OR 0.74 *p* = 0.51).

**Conclusions:**

The present meta-analysis shows that in acute perforated diverticulitis with purulent peritonitis laparoscopic lavage is comparable to sigmoid resection in term of mortality but it is associated with a significantly higher rate of reoperations and a higher rate of intra-abdominal abscess. No differences in term of mortality were demonstrated at follow-up. Further studies are needed to better define the safety and appropriateness of this treatment.

## Background

Left colon diverticulosis is a common disease in western countries with an increasing prevalence due probably to the lifestyle [1]. Its prevalence is estimated at 5 % at 40 y.o. and increases with the ages till the 80 % in the elderly [2, 3]. The commonest complication is acute diverticulitis and recent evidences reported a lifetime risk to develop diverticulitis in 4 % of patients [4]. Acute diverticulitis’ treatment depends on the severity of the inflammation, graded with several proposed scoring systems, all based on the CT scan findings [5–8] and it varies from medical therapy with or without antibiotics in mild inflammation, percutaneous drainage or sigmoid resection in diffuse peritonitis. Up to 25 % of patients with acute diverticulitis requires emergency surgery due to the disease severity [3, 9] and traditionally the treatment was the Hartmann procedure, with high morbidity (30–50 %) and mortality (10–20 %) associated [10, 11]. Due to the high morbidity and mortality of the procedure and in order to avoid a stoma, laparoscopic lavage has been proposed for perforated diverticulitis with purulent peritonitis as a lesser invasive treatment [12]. The first report of this technique in a prospective cohort of patients demonstrated that laparoscopic lavage was safe and feasible with very low mortality and morbidity rate (3 and 4 % respectively). Since its publication there was a great debate among the scientific community about this new treatment and randomized controlled trials have been launched to better investigate the issue. The aim of the present meta-analysis is to investigate the safety and the feasibility of laparoscopic lavage compared to sigmoid reection in perforated acute diverticulitis with purulent peritonitis.

## Material and methods

### Literature search strategy and studies selection

A systematic research was performed independently by two different investigators (MC and FCo) in Medline, Embase, PubMed, Cochrane Central Register of Controlled Trials (CCTR) and Cochrane Database of Systematic Reviews (CDSR) until March 2016. The search terms were: “laparoscopic lavage”, “diverticulitis”, “perforated diverticulitis” combined with AND/OR. No search restrictions were imposed. The references of selected articles were also reviewed. Duplicate published trials were considered only in the last or at least in the more complete version. All the retrieved articles were selected if they met the inclusion criteria.

### Selection criteria

For this metaanalysis were selected prospective clinical trials including patients with suspected perforated purulent diverticulitis that underwent surgical intervention and were randomized to receive bowel resection or laparoscopic lavage. Case reports, letters, reviews and metaanalysis, retrospective studies and non English language publications were excluded.

### Data extraction, outcome measures

Data were extracted for the intention to treat analysis or the modified intention to treat according to the inclusion criteria of the present meta-analysis. For each selected study were reported the following data: year of publication, study's characteristics, inclusion and exclusion criteria, patients’ characteristics, sample size, type of intervention, length of stay, length of surgery, reoperation at index admission, mortality, specific morbidity at index admission (intra abdominal abscess, UTIs, pneumonia, wound infections, heart and lung complications), severe morbidity and mortality at 90 days, mortality, reoperations and presence of stoma at 12 months.

#### Assessment of risk of bias

There is a potential risk of overestimating the beneficial treatment effects of RCT with a resultant risk of bias. The risk of bias was assessed comprehensively according to guidelines of The Cochrane Collaboration [13] and six items have been considered relevant (Table 1): 1) whether the method of allocation was truly random; 2) whether there was proper allocation concealment; 3) whether the groups were similar at baseline; 4) whether the eligibility criteria were documented; 5) whether loss to follow-up in each treatment arm was specified; 6) whether intention-to-treat analysis was conducted. Therefore the evaluation of the quality level of the study was conducted as follows: Positive answer to at least six questions was required for a trial to be rated as high quality. With a positive answer to five or four questions the study was considered of fair quality. With a positive answer to three or fewer questions the study was registered as low quality.

**Table 1:** Studies’ quality

Study id	Arndomization	Allocation concealment	Homogeneous baseline characteristics	Elegibility criteria	Lost at follow-up and drop-outs described	Intention to treat analysis	Study quality
DILALA 2016	Yes	Yes	Yes	Yes	Yes	Yes	High
SCANDIV 2015	Yes	Yes	Yes	Yes	Yes	Yes	High
LADIES 2015	Yes	Yes	Yes	Yes	Yes	Yes	High

### Statistical analysis

Data were analyzed with Review Manager (RevMan) (Version 5.3 Copenhagen: The Nordic Cochrane Centre, The Cochrane Collaboration, 2011). Outcomes were expressed as weighted Odds Ratio (OR) and 95 % confidence interval (95 % C.I.) and were calculated with the fixed-effects and random-effects models [14, 15]; statistical heterogeneity was quantified using the I^2^ inconsistency test and if significant (*p* <0,1) were reported only the results of the random-effects model.

## Results

### Studies selection

**Fig. 1:**
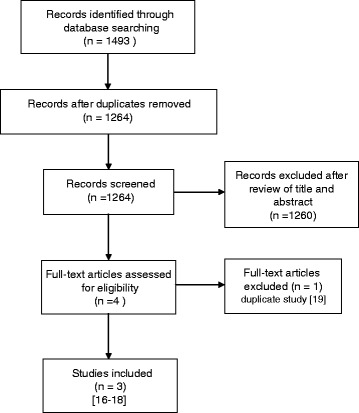
PRISMA flow diagram

One thousand two hundred sixty-four abstracts were found and after a first review four were selected and identified as potentially eligible for our study [16–19]; among them, after a full review of the manuscripts, one study was excluded because was the publication of preliminary results of an included study [19]. For the LADIES trial [17] were included patients of the LOLA arm. PRISMA flow diagram is shown in Fig. 1.

### Quality of trials and studies characteristics

There was good agreement between the reviewers (MC and FC) about the eligibility and quality of the studies. Table 1 demonstrates the quality of the three included RCT].

Table 2 summarizes studies’ characteristics.

**Table 2:** Studies’ characteristics

Study	Year	Country	Study design	Inclusion criteria	Exclusion criteria	Primary outcome	Sample size	Elegible patients	Analysis	Enrolled patients	Laparoscopic drainage	Bowel resection	Study quality	Limits
DILALA	2010–2014	Sweden, Denmark	multicentric RCT	patients with Hinchey III diverticulitis at diagnostic laparoscopy	not reported	reoperation at 12 months	64	not reported	ITT	83	43	40	High	
SCANDIV	2010–2014	Sweden, Norway	multicentric RCT	patients with free air at abdominal CT scan	obstruction pregnancy	severe complications and death at 90 days	130	216	modified ITT	144^a^	74^a^	70^a^	High	includes also patients with Hinchey I-II
LADIES	2010–2013	Belgium, Netherlands, Italy	multicentric RCT	patients with Hinchey III diverticulitis at diagnostic laparoscopy	dementia, previous abdominal irradiation, high dose steroidal therapy, shock, age >85	severe complications and mortality at 12 months	264	241	ITT	88	46	42	High	premature ending

^a^ subgroup analysis of patients with Hinchey grade I-II-III diverticulitis

#### Inclusion criteria

all the selected studies included patients >18 y.o. with evidence of perforated sigmoid diverticulitis at the CT scan and the indication of urgent surgery. In the LADIES [17] and the DILALA [18] trials patients were randomized after the demonstration of Hinchey III purulent diverticulitis at the diagnostic laparoscopy; in the SCANDIV [16] trial patients were randomized after the CT scan: therefore were also randomized patients with evidence of Hinchey I-II diverticulitis at laparoscopy. In all the studies patients with Hinchey IV-fecaloid peritonitis were drop out from the study and received Hartmann procedure.

#### Treatment

In all studies patients received empiric antibiotic therapy before surgery. Laparoscopic lavage was performed with at least 3–4 L of warm saline water. After the laparoscopic lavage patients received a colonoscopy after a time variable between 4 and 12 weeks but routine sigmoidectomy was not recommended. In the SCANDIV trial [16] colonic resection was performed in laparoscopy or with open surgery according to the centre/surgeon’s preference, with or without primary anastomosis; in the LADIES trial [17] patients in resection group were further randomized to receive Hartmann procedure or primary anastomosis. In the DILALA trial [18] patients randomized to resection all underwent Hartmann procedure. All the included patients had an abdominal drain after operation and were treated according to the local standards.

### Reoperation and mortality at index admission

**Fig. 2:**
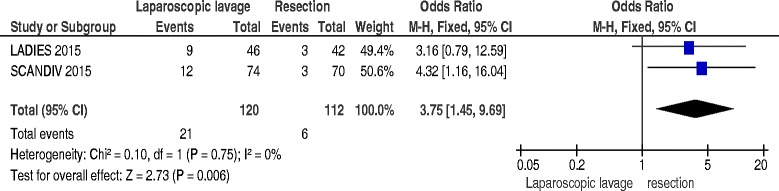
Reoperation at index admission

**Fig. 3:**
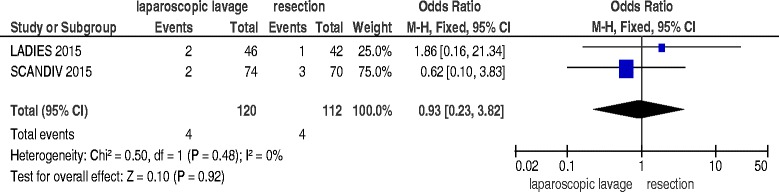
Mortality at index admission

Data about reoperation at mortality at index admission were available for two studies [16, 17] and included 232 patients: laparoscopic lavage failed, and needed a reoperation, in 17.5 % of the patients (OR 3.75; 95 % C.I. 1.45–9.69; p = 0.006) but with no significant differences in mortality (OR 0.93; 95 % C.I. 0.23–3.82; *p* = 0.92) (Figs. 2 and 3).

### Specific complications

**Fig. 4:**
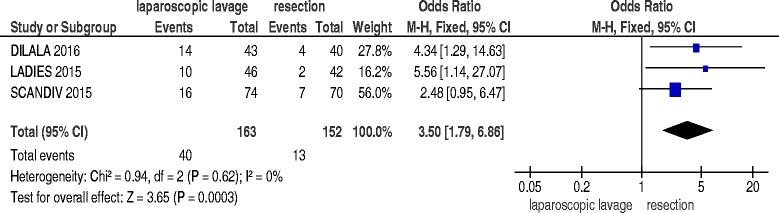
Intra-abdominal abscess

All the three studies [16–18] reported data about specific complications during the index admission, including 315 patients. Laparoscopic lavage was associated with a significantly higher incidence of intra-abdominal abscess (OR 3.50; 95 % C.I. 1.79–6.86; *p* = 0.0003) (Fig. 4), a significantly reduced incidence of wound infections (OR 0.14; 95 % C.I. 0.04–0.45; *p* = 0.0009) and no significant differences in pneumonia (OR 1.13; 95 % C.I. 0.47–2.69; *p* = 0.79), heart and lung complications (OR 0.60; 95 % C.I. 0.31–1.19; *p* = 0.15) and urinary tract infections (OR 1.20; 95 % C.I. 0.29–4.97; *p* = 0.80).

### Length of stay, length of surgery

No data in amenable format for meta-analysis were available in the three included clinical trials.

### 90 days morbidity and mortality

All the three studies reported 90 days morbidity [16–18]: the analysis included 315 patients. Laparoscopic lavage results in an increased morbidity with a subliminal statistical significance (OR 1.70; 95 % C.I. 1.00–2.87; *p* = 0.05). Data about 90 days mortality were reported in only two studies [16, 17] with 232 patients included: there were no significant differences in 90 days mortality (OR 0.83; 95 % C.I. 0.32–2.11; *p* = 0.69).

### 12 months reoperations, mortality and stoma

**Fig. 5:**
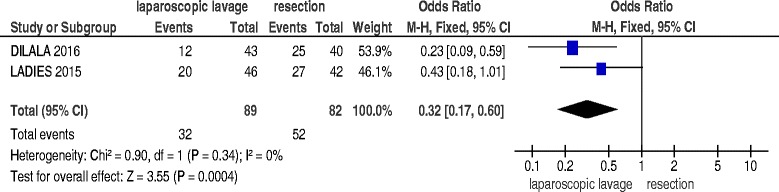
12 months reoperations

Two studies [17, 18] reported data about 12 months reoperation rate, mortality and presence of stoma, including 191 patients. Laparoscopic lavage was associated with significantly lesser reoperations (OR 0.32; 95 % C.I. 0.17–0.60; *p* = 0.0004) (Fig. 5); there were no significant differences in mortality (OR 0.74; 95 % C.I. 0.30–1.82; *p* = 0.51) and in presence of stoma (OR 0.44; 95 % C.I. 0.10–1.93; *p* = 0.27).

## Discussion

The present meta-analysis shows that laparoscopic lavage in perforate acute diverticulitis with purulent peritonitis is associated with more morbidity such intra-abdominal abscess at index admission without differences in term of mortality. Long term results show that laparoscopic lavage is associated with lesser reoperations.

The treatment of peritonitis from acute diverticulitis is an issue of great debate and great interest. Historically the management of peritonitis involved the Hartmann procedure with sigmoid resection and a terminal colonostomy. This procedure is associated with high mortality and morbidity, due above all to the patients’ characteristics; furthermore the presence of a stoma, with its impact on the quality of life, requires a further hospitalization and surgical intervention to restore intestinal continuity later in the time. Successively, laparoscopy and primary anastomosis were proposed also in acute setting and seems to be associated with better results [20, 21]. Laparoscopic lavage was proposed in 1996 [22] and since the first appearance lot of case series and review were published reporting contrasting results [23]. Despite promising results there are great debate and skepticism about this new approach to peritonitis due to the non definitive treatment of the underlying pathology [24–26].

Results of the present meta-analysis do not show significant differences in term of mortality during the index admission and during the follow up in patients with purulent peritonitis from acute diverticulitis. However, despite the subliminal significance, laparoscopic lavage is associated with an increased severe morbidity within 90 days from the event. This data is also confirmed by the elevated need of reoperations during the index admission, due to the failure of the treatment, as highlighted by the significative higher incidence of intra-abdominal abscess as a consequence of the poor source control. The presence of abscess and further reoperations do not resulted in augmented mortality; however it inevitably required prolonged antibiotic therapies, since the source of the infection was not removed, in discordance with the principles of adequate source control in treatment of sepsis and increasing the risk for antibiotic resistant pathogens selections. Laparoscopic lavage results in a reduced rate of wound infections and no differences in term of medical complications (pneumonia, UTI, heart and lung complications). In all the three trials there were no significative differences in term of length of stay but no data amenable to be meta-analyzed are available. On the other hand laparoscopic lavage, when successful, resulted in a complete resolution of the peritonitis without stoma: despite after 1 year there are no differences in presence of stoma, patients randomized to resection undergo reoperations significantly more frequently compared to those randomized to laparoscopic lavage due to restoring intestinal continuity, with no differences in term of mortality.

The results of the present meta-analysis should be interpreted at the light of some considerations and limitations. The number of included studies and patients is quite small. Furthermore the LADIES trial [17] was ended before reaching the sample size requested due to a safety issues and therefore it was largely underpowered. Moreover the included studies were not homogeneous in inclusion and exclusion criteria: the SCANDIV trial [16] randomized patients before the diagnostic laparoscopy with the inclusion in the study of Hinchey I-II patients and results could be consequently overestimated. Above all, the three included studies had different primary end points barely combinable, reducing the number of the patients and the strong of the evidence.

Even in the randomized studies, among eligible patients, only a small part of them was effectively randomized (Table 2): for sure to conduct a randomized trial in an emergency setting is really difficult but this could be a potential origin of selection bias, with only patients in better conditions selected for randomization [16].

Another randomized controlled trial is now ongoing-the LapLand trial [27]-with similar study’s design but with the operative and in-hospital mortality as primary endpoint. Enrollment was expected to be completed in December 2015 and there is a great expectation for the results. The results of the present meta-analysis are not definitive and they should be interpreted also at the light of the poor data available, awaiting for this new trial's results.

## Conclusions

In conclusion the present meta-analysis shows that in acute perforated diverticulitis with purulent peritonitis laparoscopic lavage is comparable to sigmoid resection in term of mortality but it is associated with a significantly higher rate of reoperations and a higher rate of intra-abdominal abscess. No differences in term of mortality were demonstrated at 90 days and 12 months. After 1 year from the event there were no differences in presence of stoma and patients randomized to resection underwent significantly more reoperations. Further studies are needed to better define the safety and appropriateness of this treatment.
